# Directional Bias in the Perception of Cast Shadows

**DOI:** 10.1177/2041669516682267

**Published:** 2017-01-01

**Authors:** Tomomi Koizumi, Hiroyuki Ito, Shoji Sunaga, Masaki Ogawa

**Affiliations:** Graduate School of Design, Kyushu University, Fukuoka, Japan; Faculty of Design, Kyushu University, Fukuoka, Japan; Research Center for Applied Perceptual Science, Kyushu University, Fukuoka, Japan

**Keywords:** cast shadow, shading, lighting direction, three-dimensional perception

## Abstract

Previous studies have demonstrated that the perception of shading is based upon assumptions about lighting direction, for example, light from above. However, it is not clear whether these assumptions are used in the perception of cast shadows. Moreover, it is unclear whether a perceptual interaction exists between shading and cast shadows because until now they have been studied separately. In this study, we investigated through three experiments whether the light-from-above (or another direction) assumption is used in interpreting ambiguous cast shadows, and whether shading information influences the interpretation of cast shadows. Our results indicate the existence of the light-from-above assumption in interpreting cast shadows. Consistent shading information enhanced the interpretation, and judgments of lighting direction were also based on both cast shadow and shading information. However, the perceptual determination of shape from shading was relatively independent of the cast shadow interpretation or the lighting direction judgments of the scene.

## Introduction

**Figure 1.**
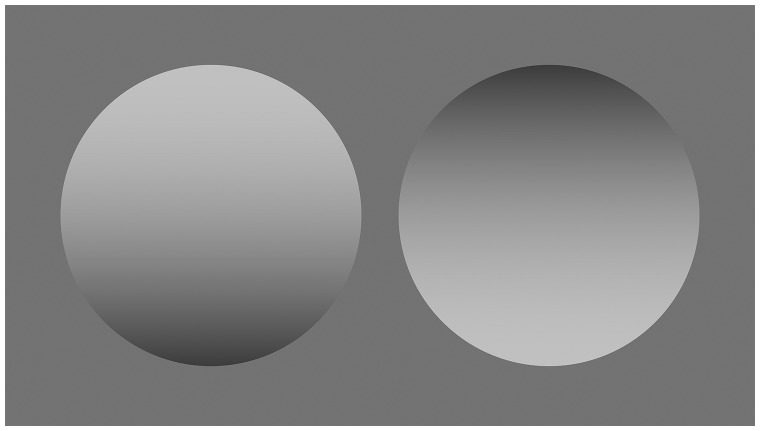
Example of a single-light-source and the light-from-above assumptions. The left disk looks convex, and the right disk looks concave. Even if the image is rotated 180° in the image plane, only a disk that is lighter on top in the observer’s head coordinates looks convex.

Humans receive spatial information from shading and shadows as depth cues. Shading is defined as “the variation of reflected light on a surface patch which faces directly the light source” (p. 289; Mamassian, Knill, & Kersten, 1998). Conversely, shadows can be classified as either attached or cast. When a part of the object surface is darker because the object itself occludes the light, this is called an attached shadow. When a shadow is produced on a distant surface, this is called a cast shadow. However, there is no reason to discriminate between shading and attached shadow for images with gradation in light strength shown on a computer screen (as shown in Figure 1). Thus, throughout this article, we refer to the gradation of light strength on the computer screen as shading.

There are known to be two constraints about a light source when we perceive shape from shading (Kleffner & Ramachandran, 1992; Ramachandran, 1988a, 1988b). One is the assumption of a single light source. For example, as shown in Figure 1, the left disk looks convex while the right disk looks concave (or, at least, not convex). It may be difficult for readers to see both disks as convex at the same time, implying two different light sources: One placed above the left disk, and the other placed below the right disk. The other is the assumption of light from (head centered) above. The readers can confirm this assumption by rotating Figure 1 180° in the image plane. Convexity is always perceived in the disk that is light at the top in a head-centric coordinate system. These two constraints may relate to the fact that the sun always shines above the horizon, typically above the head in an upright orientation. Conversely, at sunrise or sunset, light comes from the sun in a horizontal direction. However, for horizontal light, the lighting direction could be right, left, in front of, or behind an observer.

In addition to the assumption of light from above, the assumption of light from above-left in the perception of shape from shading has been studied. In Sun and Perona (1998), observers searched for a convex or concave object as a target among distractors. The light directions (i.e., shading directions) of the target and distractors were opposite. The observers could find the target the fastest when the light came from a direction that was 30° to 60° toward the left relative to the vertical. Similarly, Mamassian and Goutcher (2001) asked observers to judge whether the width of strips that looked like they were bulging was narrow or wide, which perceptually changed depending on the assumed light source direction. Their results showed that the assumed light source direction was biased 26° to the left of overhead. Both studies found that the prior assumption of the light source position was above-left, rather than directly above.

However, in the perception of cast shadows, there are very few studies on this light source assumption. Kersten, Knill, Mamassian, and Bülthoff (1996) found that the motion of a cast shadow induced the perceived three-dimensional (3D) motion of an object. Their stimuli consisted of a stationary square and a moving *cast shadow*. They showed that when the pseudo-cast shadow moved to the lower right, it caused perceived movement of the square more frequently than when moving to the upper left. These results further indicate that the light source is assumed to be stationary (see also, Kersten, Mamassian, & Knill, 1997; Mamassian et al., 1998), and that the lower right direction is relatively favored as a position of the cast shadow; that is, the upper left is the favored light source position producing the cast shadow.

One of the two purposes of this study was to investigate whether there was an assumption of light from above or from another orientation in the perception of cast shadows. If the assumption of light from above is adopted, a cast shadow may be preferred below the object. The other purpose was to confirm the perceptual interaction between shading and cast shadows (Casati, 2014). As the perception of shading and cast shadows has often been studied separately, it is unclear whether one actually affects the other. In this study, we investigated whether shading affected the perception of ambiguous cast shadows from the viewpoint of matching direction. If shading has an influence on the perception of cast shadows, the directional preference of a cast shadow should be enhanced or degraded by adding matched or unmatched shading information, respectively.

To test these hypotheses, we performed three experiments in a random order for each participant. The first one was the main experiment of the present article (Experiment A). The aims of this experiment were to examine the existence of a light-from-above (or from-another-orientation) assumption in the perception of cast shadows and to investigate the effect of shading on the perceived correspondence between the disk and cast shadows, for example, whether they were mutually independent, additive, or subtractive. The second experiment examined the perceived direction of the light source (Experiment B). The aims of this experiment were to determine whether there was any light-direction bias in direct light-direction judgments and to confirm that the judged light directions were consistent with the cast shadow interpretation in Experiment A. The third experiment investigated the perceived shape of the object surface (Experiment C). The aims of this experiment were to determine whether there was any directional bias in interpreting shape from shading in the display used in the present experiments, to confirm that the perceived shape from shading was consistent with the judged light directions, and to test whether there was a relationship between the cast shadow interpretation and the perceived shape from shading.

We presented pseudo shadows on both sides of a disk in either a vertical or horizontal orientation. When the distances to the both-sides shadows from the disk are the same, the disk-shadow correspondence should be stronger for the side that was opposite to the assumed light direction. In addition, it is possible to determine a distance ratio of two cast shadows on opposite sides of a disk where the strength of the disk-shadow correspondence in both directions was balanced, that is, the directional bias was canceled by additional distance. Thus, the distance ratio indicates the strength of the directional bias in the disk-shadow correspondence in Experiment A.

## General Design

### Participants

Seven male and nine female volunteers with normal or corrected-to-normal visual acuity took part in the experiments, after having signed informed consent regarding their participation. They ranged in age from 19 to 27 years (*M*: 22.06; *SD*: 1.63). All participants received a book coupon for 500 yen as recompense. They were naive to the purpose of the experiments.

### Apparatus

The experiments were conducted in a darkened room. We used a computer (Apple, MacBook Pro) and presented stimulus images on an LCD monitor (EIZO, FlexScanSX2462W). The size of the screen was 24.1 in., and the resolution was 1920 × 1200 pixels. We placed a black masking sheet of paper on this screen, with a circular window 32 cm in diameter. Participants observed the stimuli that were visible in the circular window with a single eye. The viewing distance was 60 cm. Head movements were restricted by a chinrest. In Experiment C only, participants used a tablet computer (Wacom, Cintiq Companion) as a response device.

### Stimuli

**Figure 2.**
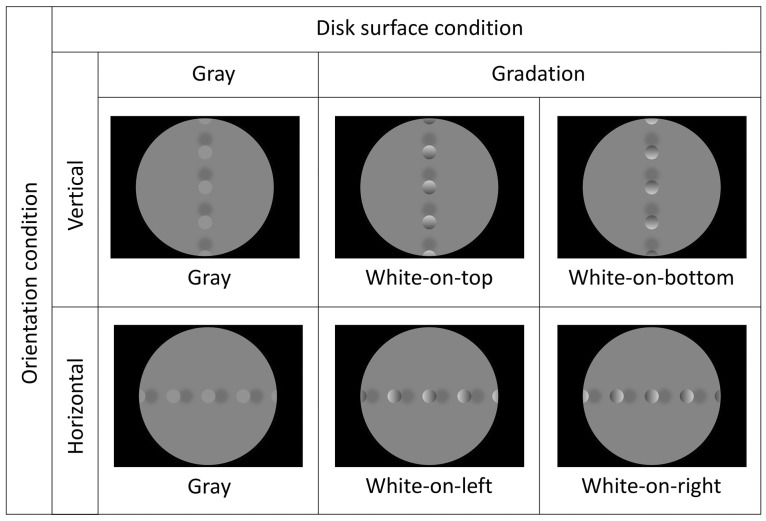
Examples of stimuli. There were two variables: orientation and disk surface. The orientation was either vertical or horizontal. The disk surface was either gray or in gradation from white to black.

Figure 2 shows examples of stimuli and conditions. Stimulus images composed of five disks and four pseudo-cast shadows (21.0 cd/m^2^) were presented on a gray background (26.5 cd/m^2^). The size of the disk was 3.2 cm in diameter, and the size of the cast shadow was about 3.4 cm in diameter as the edges of the shadows were blurred. The distance between the centers of adjacent disks was 8.2 cm, and the distance between the centers of adjacent cast shadows was the same. Cast shadows were arranged between disks. The participants were told to regard the gray background as a vertical wall. As we noticed that the background could be perceived as a ground plane, rather than a wall, to unify the participants’ interpretation, we imposed the vertical wall interpretation on the participants.

**Figure 3.**
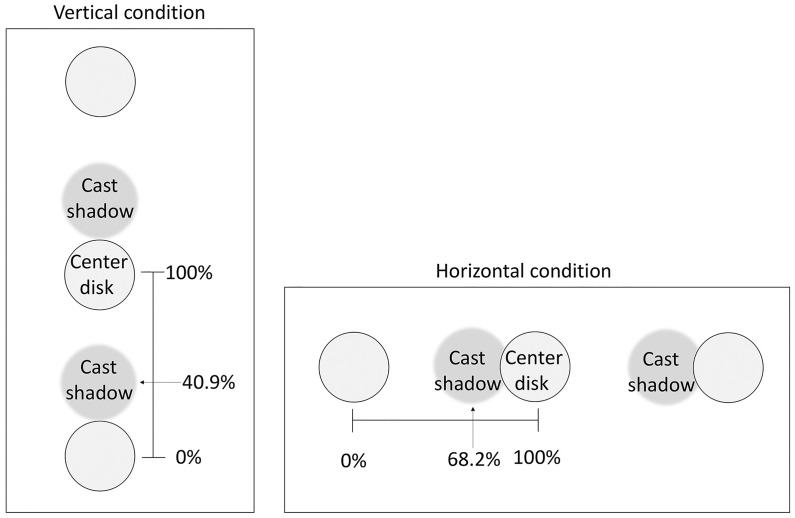
Notation of presented positions of cast shadows. The distance between disks was divided into 22 equal steps, and a cast shadow was presented at one of these positions on each trial. The figure shows schematic illustrations of cast shadows presented at the “40.9%” position in the vertical condition (left panel) and the “68.2%” position in the horizontal condition (right panel). Note that, in the vertical conditions, the cast shadow appeared above and below the center disk, while in the horizontal conditions, the cast shadow appeared left and right of the center disk.

There were two orientation conditions. Disks and cast shadows were arranged in either a vertical or a horizontal line. We assumed that a lighting bias in any direction could be measured, decomposed in the two axes. There were three disk surface conditions: two gradation (from 14.8 cd/m^2^ to 79.0 cd/m^2^) and one plain gray (46.5 cd/m^2^) conditions. In the gradation conditions, the disks had a vertical luminance gradient (white-on-top or white-on-bottom) in the vertical condition, and a horizontal luminance gradient (white-on-left or white-on-right) in the horizontal condition. Thus, there were six conditions in total. The distance between adjacent disks was divided into 22 steps, and cast shadows were arranged at one of these positions (Figure 3). The position of a cast shadow was represented as the proportion of distance between adjacent disks. Positions of 0% and 100% denote the center positions of the disks, and 50% denotes the position physically midway between the disks. We regarded the 100% shadow position as the center of the central disk, and 0% as the center of the disk below or to the left of the central disk.

## Experiment A: Judgment of Corresponding Cast Shadows

### Stimuli

The positions of the cast shadows in the stimulus images were varied in 11 steps: 4.5%, 13.6%, 22.7%, 31.8%, 40.9%, 50.0%, 59.1%, 68.2%, 77.3%, 86.4%, and 95.5%.

### Procedure

First, a fixation cross was presented in the center of the screen. Then, a stimulus image was presented for 0.5 seconds, followed by another fixation cross presentation. Finally, participants chose a cast shadow that was perceptually cast by the central disk, from two choices presented on both sides of the central disk. For example, in the vertical condition, they chose either the shadow positioned above or that positioned below the central disk. They performed the trials under the six (Three Disk Surfaces × Two Orientations) conditions in a random order. The method of constant stimuli was used to figure out the point of subjective equality in the corresponding strength to the both-sides cast shadows; 11 cast-shadow position stimuli were presented in a random order. The participants repeated this sequence 40 times; that is, 440 trials were performed per condition.

The probabilities of the chosen cast shadows were calculated and fitted with a logistic function for each condition. We calculated the shadow position at which a cast shadow on each side was chosen at 50% probability.

### Results

**Figure 4.**
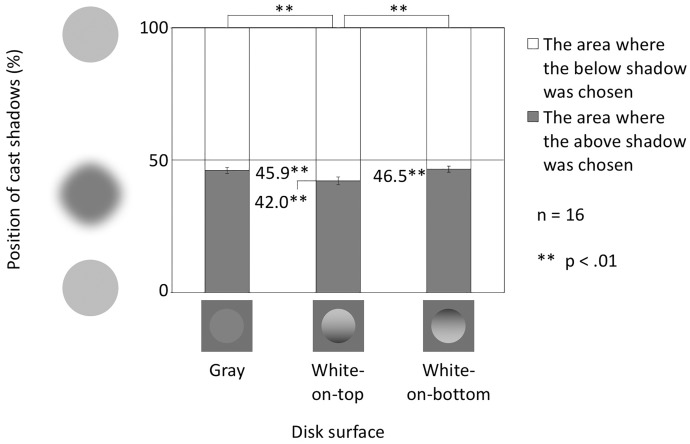
Balanced positions in the vertical conditions. The vertical axis shows the position of the cast shadows, described as the proportion of the position of the cast shadow in distance between disks. Error bars indicate *SE*s.

Figure 4 shows the vertically balanced shadow positions in the vertical conditions. The white bar shows the area where the lower shadow was chosen, and the gray bar shows the area where the upper shadow was chosen. The boundary of the bars in each condition shows the vertical balance points in the perceptual correspondence between a disk and the cast shadows on both sides of the disks. The balanced positions were 45.9% in the gray condition, 42.0% in the white-on-top condition, and 46.5% in the white-on-bottom condition.

To examine the existence of the directional bias in the chosen cast shadows, the differences between 50% and these balanced points were subjected to *t* tests. The differences were significant in all conditions. The greatest effect size (Cohen’s *d*) was found in the white-on-top condition. (Gray condition: *t*(15) = − 3.56, *p* = .0029, *d* = 0.8889; White-on-top condition: *t*(15) = −5.58, *p* = .0001, *d* = 1.3939; White-on-bottom condition: *t*(15) = −2.95, *p* = .0098, *d* = 0.7386). These results suggest that the lower shadow is more strongly connected to the center disk than the upper shadow in all vertical disk conditions, that is, participants used the light-from-above assumption when interpreting cast shadows. Even without shading information, that is, in the gray disk condition, this effect is clear.

The differences between the disk surface conditions were subjected to a one-way analysis of variance (ANOVA). The main effect of disk surface condition was significant, *F*(2, 30) = 13.07, *p* = .0001, η_p_^2 ^= 0.4658. Post hoc tests (Ryan's method, alpha levels of .05 and .01) revealed that the difference between the gray and the white-on-top conditions was significant (*p* < .01) and the difference between the white-on-top and the white-on-bottom conditions was significant (*p* < .01). The difference between the gray and the white-on-bottom conditions was not significant (*p* > .05). These results suggest that the shading information influenced the interpretation of cast shadows; in particular, the white-on-top condition enhanced the perceptual connection between the disk and the lower shadow.

**Figure 5.**
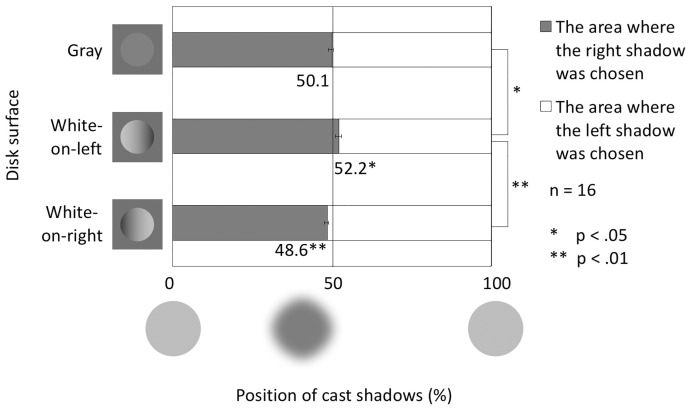
Balanced points in the horizontal conditions. The vertical axis shows the disk surface condition. The horizontal axis shows the position of the cast shadows. Error bars indicate *SE*s.

Figure 5 shows the balanced positions in the horizontal conditions. The gray bar shows the area where the right shadow was chosen, and the white bar shows the area where the left shadow was chosen. The horizontally balanced position was 50.1% for the gray condition, indicating little directional bias in the horizontal dimension. However, in the white-on-left and white-on-right conditions, a small bias was found toward the opposite direction of the white part of the disk, that is, right (52.2%) or left (48.6%), respectively.

In the same way as for the vertical conditions, *t* tests were performed. The difference in the gray condition was not significant, *t*(15) = 0.23, *p* = .8231, *d* = 0.0569. This indicates that the horizontal component of the possible light-direction assumption was not large enough to be found in the gray condition. The differences in the gradation conditions were significant (White-on-left condition: *t*(15) = 2.78, *p* = .0140, *d* = 0.6955; White-on-right condition: *t*(15) = −3.13, *p* = .0069, *d* = 0.7823). These results confirm that the right shadow was favored in the white-on-left condition and vice versa. The shading enhanced the connection between the disk and the cast shadows on the darker side of the disk.

A one-way ANOVA revealed that the main effect of disk surface condition was significant, *F*(2, 30) = 10.73, *p* = .0003, η_p_^2 ^= 0.4171. Post hoc tests (alpha levels of .05 and .01) revealed that the difference between the gray and the white-on-left conditions was significant (*p* < .05), and that the difference between the white-on-left and the white-on-right conditions was significant (*p* < .01), but that the difference between the gray and the white-on-right conditions did not reach significance (*p* > .05).

In short, our results show that the light-from-above assumption was used in interpreting cast shadows, and that shading information enhanced the connection between the disk and the cast shadow on the darker side of the disk in the vertical and horizontal conditions.

## Discussion

We confirmed that the light-from-above assumption was used in the perception of cast shadows in all vertical conditions. In the white-on-top condition, the perceptual connection between the disk and the shadow below was stronger than that in the gray condition. A reasonable explanation of this result is that shading information indicates the direction of light on the lighter side of the gradation disk; that is, the white-on-top disk enhanced the light-from-above assumption. However, in the white-on-bottom condition, shading information did not affect the results. In the white-on-top condition, as the light-from-above assumption and the light direction suggested by the shading information were consistent, both effects were present and additive. However, in the white-on-bottom condition, as the light-from-above assumption and the light direction suggested by the shading information were not consistent, the latter might be ignored in the interpretation, based on the single-light-source assumption. Casati (2014) observed that cast shadows are independently processed from attached shadows. When we assume that the two processes are independent at the first level of processing and integrated at the second level, our results are consistent with Casati (2014).

In the horizontal condition, we could not find a directional bias in the gray disk condition; the preferred cast shadows depended on the physical distance from the disk. When the preferred light direction for cast shadows is above-left, as suggested by Mamassian and Goutcher (2001) in shape from shading, the light-from-left bias could be observed in the horizontal condition. One possible reason for this result is that the stimulus arrangement was only in a left-to-right (horizontal) direction in our stimulus images. Thus, a light-from-left component in a possible light-from-above-left bias might be underestimated. Another possible reason is that the participants who underwent the gray disk conditions after the gradation disk conditions tended to rely more on the shading information. We are planning to measure the bias only with plain disk stimuli but aligned in varied orientations in the next research project.

We assume that the light direction suggested by shading is defined by the lighter side of the shading. This means that disks with shading are assumed to be convex, either because of a general preference for convexity or scene familiarity, for example, flying balls are more likely to exist than flying bowls. In addition, cast shadows could enhance the flying ball perception. In Experiment C, we confirmed this convex bias, and we discuss this issue later on.

**Figure 6.**
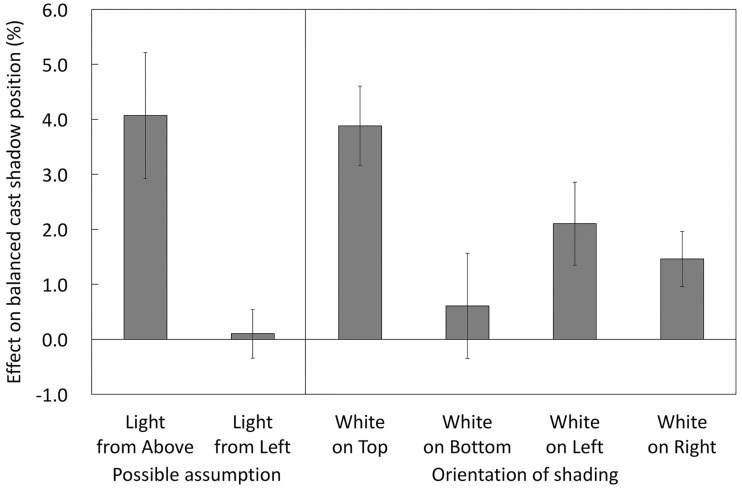
Effects of the factors on the directional bias in cast shadow interpretation. The directional bias caused by each factor was calculated. “Light-from-Above/Left assumption” is the difference in absolute value between 50% and the results from the gray disk condition in the vertical or horizontal arrangement, respectively. “White-on-Top/Bottom” is the difference in absolute value between the results from the white-on-top or bottom and the results from the gray disk conditions in the vertical arrangement. “White-on-Left/Right” is the difference in absolute value between the results from the white-on-left or right and the results from the gray disk conditions in the horizontal arrangement. Error bars indicate *SE*s.

Figure 6 shows the magnitudes of the effects caused by the factors tested here on the directional bias in cast shadow interpretation. The light-from-above assumption caused 4.1% of the shift in the balanced position, while the possible light-from-left component in the light direction assumption caused only a 0.1% shift. The white-on-top shading also caused 3.9% of the shift, while the white-on-bottom shading had a much smaller effect (0.6%). In the vertical arrangement, while the light-from-above assumption was effective in interpreting cast shadows, the white-on-top shading added nearly the same amount of effect to the cast shadow interpretation. Thus, the light-from-above assumption directly affects the interpretation of cast shadows and indirectly affects them through the enhancement of shading information. However, the white-on-left and white-on-right shadings caused moderate effects (2.1% and 1.5%, respectively). In the horizontal arrangement, in addition to the small directional bias in the cast shadow interpretation as shown by the gray condition, the effects of shading were about half as large as those in the vertical arrangement.

## Experiment B: Judgment of Lighting Direction

### Stimuli

The positions of the cast shadows in the stimulus images were varied in eight steps: 9.1%, 22.7%, 36.4%, 50.0%, 63.6%, 77.3%, 90.9%, and 100% (0%). In addition, we used a stimulus image that had no cast shadows. The disk surface conditions were the same as those in the previously described experiment.

### Response Screen

We made a response screen to allow our participants to choose any lighting direction. A disk was presented in the center of the screen, with 36 lines and numbers (from 1 to 36) radiating out from the center of the disk. Participants chose a number corresponding to the direction from which they perceived the central disk to be lit.

### Procedure

After presentation of a fixation cross, a stimulus image was presented for 0.5 seconds, followed by the response screen presentation. Participants chose a number that indicated the corresponding lighting direction. In addition, they reported the degree of conviction on a six-degree scale (0–5). For example, 0 indicated *no idea*, 1 indicated *the weakest conviction*, and 5 indicated *the strongest conviction*. They performed 54 trials (9 positions × 3 disk surfaces × 2 orientations) in a random order after six practice trials.

### Results and Discussion

We analyzed only vertical and horizontal lighting direction judgments because the other lighting directions were rarely chosen and, in that case, conviction degrees were very low throughout all stimulus conditions. There may be two reasons for these limited responses: One is that the shading and cast shadows always provided lighting direction cues within the same dimension, that is, vertical or horizontal. It would be worth testing a condition where horizontal shading and vertically placed cast shadows are combined. The other is that the disks and cast shadows were always aligned in either a vertical or horizontal line.

**Figure 7.**
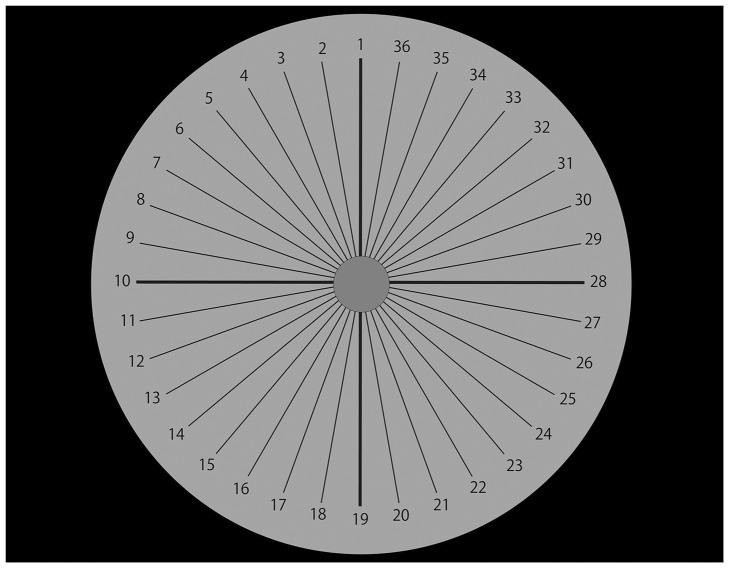
Response screen in Experiment B. Participants chose a number corresponding to one of 36 directions from which they perceived the central disk to be lit.

For the vertical directions (1 and 19 in Figure 7), the conviction degrees were signed as positive for “above (1)” responses and signed as negative for “below (19)” responses. Other responses were scored zero. Similarly, for the horizontal directions (10 and 28 in Figure 7), the conviction degrees were signed as positive for “left (10)” responses and signed as negative for “right (28)” responses. Other responses were scored zero.

**Figure 8.**
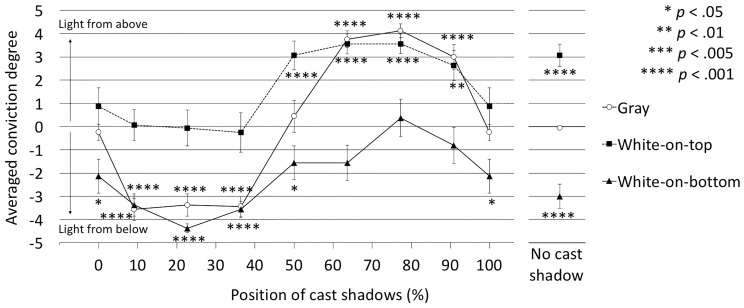
Judged lighting directions with degrees of conviction in the vertical conditions. The positive area corresponds to light from above, and the negative area corresponds to light from below. *p* indicates the probability of the difference between each data point and 0. Error bars indicate *SE*s.

Figure 8 shows the judged lighting directions with averaged degrees of conviction for each cast shadow position in the vertical conditions. A two-way ANOVA revealed that the main effects of disk surface, *F*(2, 30) = 28.88, *p* < .0001, η_p_^2 ^= 0.6581, and cast shadow position, *F*(8, 120) = 39.89, *p* < .0001, η_p_^2 ^= 0.7267, and the interaction between them, *F*(16, 240) = 5.21, *p* < .0001, η_p_^2 ^= 0.2579, were significant.

To examine the directional bias in the perceptual lighting direction, the differences between the averaged conviction degrees and 0 were subjected to *t* tests. The results are shown in Figure 8. In the gray condition, in the 50% and 100% (0%) positions, and in the no-cast-shadow conditions, little directional bias was found. Positive and negative values, that is, dominance of light-from-above and light-from-below judgments, switched at 50% shadow position. This result means that the lighting directions were suggested solely by cast shadow positions, that is, whether closer shadows were below or above the disk. These results were not completely consistent with the results of Experiment A, which showed a light-from-above (below-shadow) bias.

In the white-on-top condition, the light-from-above judgments were dominant in the positions between 50% and 90.9%, and in the no-cast-shadow case. In these shadow positions, there was no contradiction in the lighting direction information between cast shadow and shading. The light directions in the other positions were ambiguous, that is, not significantly different from 0. In these shadow positions, as the light-from-below judgments were dominant for the plain gray disk conditions, it is plausible that the lighting direction information from the cast shadow positions and the white-on-top shading canceled out or rivaled each other.

Conversely, in the white-on-bottom condition, the dominance of the light-from-below judgments was significant in the positions between 9.1% and 50%, 100% (0%), and in the no-cast-shadow condition. The light directions in the other positions were ambiguous. As shown in the white-on-top conditions, in these shadow position conditions, there may be a rivalry in the judgments of lighting direction because of the contradiction between the cast shadow and the shading information.

**Figure 9.**
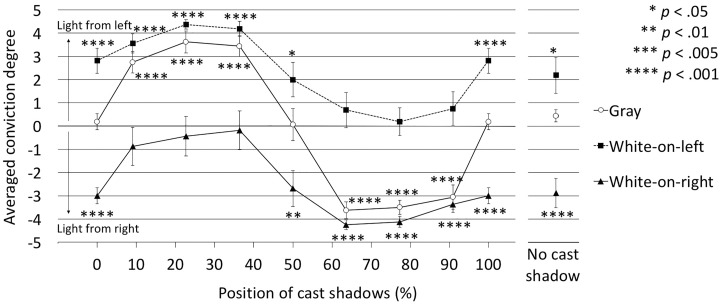
Judged lighting directions with degrees of conviction in the horizontal conditions. The positive area indicates the dominance of light-from-left judgments, and the negative area indicates the dominance of light-from-right judgments. Error bars indicate *SE*s.

Figure 9 shows the judged lighting directions with averaged degrees of conviction for each cast shadow position in the horizontal conditions. A two-way ANOVA revealed that the main effects of disk surface, *F*(2, 30) = 53.64, *p* < .0001, η_p_^2 ^= 0.7815, and cast shadow position, *F*(8, 120) = 27.76, *p* < .0001, η_p_^2 ^= 0.6492, and the interaction between them, *F*(16, 240) = 3.56, *p* < .0001, η_p_^2 ^= 0.1920, were significant.

We performed the same *t* tests as those for the vertical conditions. In the gray condition, the light-from-left judgments were dominant in the positions between 9.1% and 36.4%, while the light-from-right judgments were dominant in the positions between 63.6% and 90.9%. Simply, the vicinity between the center disk and cast shadows seems to determine the perceived lighting direction. In the other positions, the light direction was ambiguous.

In the white-on-left condition, the light-from-left judgments were dominant in the positions between 9.1% and 50.0%, 100.0%, and in the no-cast-shadow conditions. Conversely, in the white-on-right condition, the light-from-right judgments were dominant in the positions between 50% and 100%, and in the no-cast-shadow conditions. In both white-on-left and white-on-right conditions, when the information from the cast shadow positions and shading contradicted, the lighting direction judgments were ambiguous, as in the vertical conditions.

Taken together, the results from the vertical and horizontal conditions indicate that the cast shadow position was strongly involved in the judgment of the lighting direction in all disk conditions, and that the lighter side directions were strongly involved in the gradation conditions. We confirmed that the averaged degree of conviction of light direction judgment fluctuated between both cues in the gradation conditions, that is, when both cues to the light direction contradicted, conflicts in judgments arose. It is unclear whether the conviction of the lighting direction becomes stronger if cast shadows and shading suggest the same lighting direction in the gradation condition because of a ceiling effect.

We did not find a clear bias toward light-from-above judgments. In the white-on-bottom condition, light-from-below judgments were dominant around the 50% position, where in Experiment A, we found the bias that a disk and a cast shadow presented below it were perceptually connected. Thus, it is plausible that cast shadow interpretation precedes light direction judgments based on shading information.

## Experiment C: Shape of the Disk Surface

### Stimuli

The stimulus images were the same as those in Experiment B.

### Response Screen

We used a pen-tablet computer (Wacom, Cintiq Companion) with which participants made their responses. They were required to draw lines with the pen to indicate the perceived 3D disk shape (e.g., convex or concave), and the perceived spatial relationship between the disk and the background (e.g., floating or attached), in a top view and a side view. Participants explained their response when they perceived the disk surface as a complex shape.

### Procedure

After presentation of a fixation cross, a stimulus image was presented for 0.5 seconds. Then, the fixation cross was presented again. Finally, participants drew the perceived 3D shape of the disk surface and the background. They were able to observe stimulus images any number of times. They performed 54 trials, that is, 9 cast shadow positions (including no-cast-shadow condition) × 2 orientation (vertical or horizontal) × 3 disk surfaces (gray and two gradation conditions) in a random order after a practice of six trials.

### Results and Discussion

We classified participants’ responses into four types of shapes of disk surface: convex, concave, plane, and others. Table 1 shows the number of participants who perceived the disk surface as each shape.

**Table 1.** Classification of the Shape and Numbers of Corresponding Participants (*n* = 16) in Each Condition.

			Position of cast shadows								
Alignment	Disk surface	Shape	9.1%	22.7%	36.4%	50.0%	63.6%	77.3%	90.9%	100%	No cast shadow
Vertical	Gray	Convex	3	2	4	3	2	3	2	2	1
	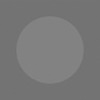	Concave	0	1	0	0	0	0	1	2	0
		Plane	13	12	12	12	13	11	12	11	15
		Others	0	1	0	1	1	2	1	1	0
	
	White-on-top	Convex	15	13	15	16	15	15	16	16	14
	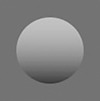	Concave	1	2	1	0	1	0	0	0	2
		Plane	0	1	0	0	0	0	0	0	0
		Others	0	0	0	0	0	1	0	0	0
	
	White-on-bottom	Convex	15	15	16	14	13	13	12	14	11
	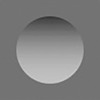	Concave	0	0	0	1	1	2	3	2	4
		Plane	0	1	0	0	1	0	0	0	0
		Others	1	0	0	1	1	1	1	0	1

Horizontal	Gray	Convex	3	3	3	4	4	3	3	1	1
	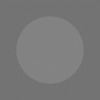	Concave	0	0	0	0	0	0	1	2	1
		Plane	13	13	13	12	12	13	12	12	13
		Others	0	0	0	0	0	0	0	1	1
	
	White-on-left	Convex	16	16	16	15	14	13	14	14	14
	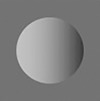	Concave	0	0	0	0	1	2	2	2	2
		Plane	0	0	0	0	0	0	0	0	0
		Others	0	0	0	1	1	1	0	0	0
	
	White-on-right	Convex	12	14	14	15	16	13	15	13	9
	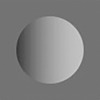	Concave	2	1	1	0	0	0	0	2	7
		Plane	1	0	0	1	0	1	0	0	0
		Others	1	1	1	0	0	2	1	1	0

It is obvious that the gray surfaces were mainly perceived as planes, irrespective of the alignment orientations and the positions of the cast shadows. At the same time, a convex shape was reported by a few participants across all the cast shadow position conditions, while a concave shape was rarely reported.

Conversely, surfaces in almost all gradation conditions were perceived as convex. Cochran’s Q tests revealed that there were no significant differences in the numbers of convex reports between the shadow position conditions, including the no-cast-shadow condition, in the white-on-top (χ^2 ^= 13.9130, *df* = 8, *p* = .0841), white-on-bottom (χ^2 ^= 11.8033, *df* = 8, *p* = .1602), and white-on-left (χ^2 ^= 10.0000, *df* = 8, *p* = .2650) conditions, but that there was a significant difference in the white-on-right (χ^2 ^= 18.9538, *df* = 8, *p* = .0151) condition. In the no-cast-shadow white-on-right condition, the number of *convex* reports was lower than in the other shadow-position conditions, and 7 out of 16 participants even reported *concave*. However, when a cast shadow was presented, the convex responses tended to increase. For example, in the white-on-right condition, compared with the no-cast-shadow condition, convex responses significantly increased in the 63.6% and 90.9% cast shadow position conditions (*p* = .0156 and *p* = .0313, respectively; McNemar’s test). The existence of a cast shadow may suppress the concave impression of the disk by suggesting that the disk is floating on the background plane. A concave shape is generally a part of the surface of a large object, not an object shape itself. Perceptual detachment of the disk from the background may contribute to the suppression of the concave impression, and cast shadows may affect the perception of shapes from shading in this limited manner.

We found that convex was chosen as the perceived shape of a disk with shading in almost all conditions as shown in Table 1. The results are not predicted from the light-from-above assumption that suggests *concave* in the white-on-bottom condition. We suggest three reasons why the white-on-bottom disks did not tend to appear as a concave shape. First, all disks in the stimulus image had the same gradation direction. In previous studies, stimulus images used in experiments included disks with gradations in both directions on the same screen. Second, convex superiority might dominate in this experiment; that is, we tend to regard objects as convex because there are few concave objects in the world. Third, cast shadows disturb the perception of a concave shape, as noted earlier.

However, this convex-biased determination of shape from shading contradicts the results from Experiment B. From the viewpoint of determining shape from shading, the convex perception means that the light comes from the lighter side of the disk. As the convex superiority was constant across the cast shadow position conditions, the lighting direction was always assumed to be the lighter side direction. However, in Experiment B, judged lighting directions were the combined or a compromise of the directions between the shading direction and the cast-shadow-position information. It seems that participants judged the lighting direction using all the information available in the scene in Experiment B, although they judged the shape from the shading with a convexity bias and almost ignored the cast shadow position in Experiment C.

## General Discussion

We performed three experiments. Experiment A showed that the light-from-above assumption was used in interpreting cast shadows, and that shading information influenced the interpretation of cast shadows. Experiment B showed that the lighting direction was judged depending both on the position of the cast shadow and on the direction of the lighter part of the disk. Experiment C showed that the disk surface was mainly perceived as a plane shape in the gray conditions, and as a convex shape in the gradation conditions, irrespective of the shadow position.

In this article, we have referred to some assumptions in perceiving shape from shading, for example, the light-from-above or single-light-source assumption (e.g., Ramachandran, 1988a, 1988b). In addition, we have also referred to a preference, that is, the convexity preference (e.g., Hill & Bruce, 1994). *Assumption* and *preference* are similar here in the point that both make a bias in perceptual interpretation, apart from their concepts. Although the light-from-above assumption has been well established in previous studies, the assumption was totally overturned by the convexity preference in Experiment C. As a result, lighting direction information from shading was incidentally determined; that is, the light may come from the bright side of the shading. The lighting direction suggested by shading also affected cast shadow interpretation, as shown in the shaded disk conditions in Experiment A. We saw another effect of the light-from-above assumption in determining shape from shading in the right panel of Figure 6. The effects of shading on cast shadow interpretation were the strongest in the white-on-top condition and the weakest in the white-on-bottom condition, with the white-on-left and white-on-right conditions in between. Thus, we think that although perceptual convexity when determining shape from shading was qualitatively determined by the convexity preference, the strength of the convexity impression (and the conviction of the lighting direction) still depended on the orientation. This dependence could result in a variable-strength effect of shading on cast shadow interpretation.

As for cast shadows, although the strength of the perceptual object–shadow connection was essentially determined by the relative distance between them, the light-from-above assumption was effective, as shown in the plain gray disk condition in Experiment A. To be precise, the matching bias toward a shadow below should be expressed as the shadow-below bias, not the light-from-above bias, because a bias toward the light-from-above judgment was not obtained in the plain gray disk condition in Experiment B. It is possible that object–shadow matching and lighting direction judgments are different tasks for the brain, in spite of the close relationship between them. In Experiment B, we confirmed that the lighting direction judgments were determined by combined or a compromise between the information from cast shadow position and shading direction. Shading may indirectly affect object–shadow matching via suggesting a certain lighting direction.

In all the experiments presented in this article, as the shading direction was always the same for all disks within one stimulus image on the screen, the single-light-source assumption was not an important factor for determining shape from shading. However, when a cast shadow exists closely to the bright side of an object, the single-light-source assumption and convexity preference may be challenged by the shadow. The result that the lighting direction judgments changed depending on the cast shadow position in Experiment B demonstrates the perceptual integration of both types of directional information, assuming a single light source. Conversely, in Experiment C, the perceptual convexity was little affected by the position of the cast shadow. These observations indicate that the single-light-source (combined with convexity) assumption may act directly within the shape-from-shading processing and act again in cognitive-level lighting-direction judgments. Therefore, it is possible that multiple light sources from perpendicular directions suggested independently by shading and shadows coexist in different levels of processing. We did not pursue this possibility in the present experiments, and it may be a direction for future research.

Another direction for future research is to investigate the meaning of *above* or *below* for a cast shadow; *above* for the perceptual determination of a shape from shading is usually considered as *above* in the head-centric frame of reference, rather than in the gravity system (Howard, Bergström, & Ohmi, 1990; Wenderoth & Hickey, 1993). This might indicate that the light-from-above assumption in determining shape from shading is implemented by a lower level process in the brain. In this study, cast shadows were observed by participants in an upright posture; that is, *above* for the head and that for gravity were identical. It is worth testing whether gravity is relevant to the cast shadow interpretation by manipulating a participant’s head orientation.

In summary, we found that the light-from-above assumption is used in object–shadow matching. Consistent shading enhanced the light-from-above interpretation of cast shadows. When judgments about lighting direction were required, information from shading and cast shadows were combined, and were used as cues to estimate the direction. Conversely, cast shadow information had little influence on the perception of shape from shading. We think that the interpretation of cast shadows and lighting direction judgments requires a scene analysis in which the perceptual (and cognitive) system gathers information from multiple cues, including shading, while the perceptual determination of shape from shading is almost independent of this process and obeys its own assumptions or preferences.
